# AI-assisted facial analysis in healthcare: From disease detection to comprehensive management

**DOI:** 10.1016/j.patter.2025.101175

**Published:** 2025-02-04

**Authors:** Chaoyu Lei, Kang Dang, Sifan Song, Zilong Wang, Sien Ping Chew, Ruitong Bian, Xichen Yang, Zhouyu Guan, Claudia Isabel Marques de Abreu Lopes, Mini Hang Wang, Richard Wai Chak Choy, Xiaoyan Hu, Kenneth Ka Hei Lai, Kelvin Kam Lung Chong, Chi Pui Pang, Xuefei Song, Jionglong Su, Xiaowei Ding, Huifang Zhou

**Affiliations:** 1 Department of Ophthalmology, Shanghai Ninth People’s Hospital, Shanghai Jiao Tong University School of Medicine, Shanghai, China; 2 Shanghai Key Laboratory of Orbital Diseases and Ocular Oncology, Shanghai, China; 3 Center for Basic Medical Research and Innovation in Visual System Diseases, Ministry of Education, Shanghai, China; 4 School of AI and Advanced Computing, XJTLU Entrepreneur College (Taicang), Xi’an Jiaotong-Liverpool University, Suzhou, China; 5 VoxelCloud Inc, Shanghai, China; 6 Microsoft Research Asia, Shanghai, China; 7 Shanghai Jiao Tong University School of Medicine, Shanghai, China; 8 United Nations University International Institute for Global Health, Kuala Lumpur, Malaysia; 9 Department of Ophthalmology and Visual Sciences, Faculty of Medicine, The Chinese University of Hong Kong, Hong Kong, China; 10 Department of Ophthalmology and Visual Sciences, The Prince of Wales Hospital, Hong Kong, China; 11 Hong Kong Eye Hospital, Hong Kong, China; 12 Eye Centre, The Chinese University of Hong Kong Medical Centre, Hong Kong, China; 13 Department of Electronic Engineering, Shanghai Jiao Tong University, Shanghai, China

**Keywords:** artificial intelligence, facial analysis, healthcare, disease screening, global health

## Abstract

Medical conditions and systemic diseases often manifest as distinct facial characteristics, making identification of these unique features crucial for disease screening. However, detecting diseases using facial photography remains challenging because of the wide variability in human facial features and disease conditions. The integration of artificial intelligence (AI) into facial analysis represents a promising frontier offering a user-friendly, non-invasive, and cost-effective screening approach. This review explores the potential of AI-assisted facial analysis for identifying subtle facial phenotypes indicative of health disorders. First, we outline the technological framework essential for effective implementation in healthcare settings. Subsequently, we focus on the role of AI-assisted facial analysis in disease screening. We further expand our examination to include applications in health monitoring, support of treatment decision-making, and disease follow-up, thereby contributing to comprehensive disease management. Despite its promise, the adoption of this technology faces several challenges, including privacy concerns, model accuracy, issues with model interpretability, biases in AI algorithms, and adherence to regulatory standards. Addressing these challenges is crucial to ensure fair and ethical use. By overcoming these hurdles, AI-assisted facial analysis can empower healthcare providers, improve patient care outcomes, and enhance global health.

## The bigger picture

Imagine a world in which a simple photograph of one’s face could offer insights that could assist doctors in detecting and managing certain diseases earlier. This possibility has emerged through the integration of artificial intelligence (AI) into facial analysis. Several health conditions leave subtle clues on our faces, indicating that AI can learn to recognize with remarkable accuracy. AI can help with early disease detection, guide treatment decisions, and track health over time by analyzing facial features. This approach has the potential to significantly improve patient care and public health worldwide. However, for this technology to be truly beneficial, we must address important issues, including data privacy, model interpretability, accuracy and fairness of AI algorithms, and compliance with digital health regulations. If these obstacles can be effectively resolved, then AI-driven facial analysis could emerge as a valuable tool in healthcare, offering advanced medical insights that are both accessible and impactful.

## Teaser

AI is changing healthcare by enabling facial analysis for health monitoring and disease screening and management. This review highlights the potential of AI-assisted facial analysis to detect subtle facial phenotypes linked to health disorders. While promising, adoption requires addressing key challenges, including privacy, interpretability, accuracy, bias, and regulation. By overcoming these hurdles, this technology can benefit patient care, offering a non-invasive, cost-effective, and user-friendly approach to improving global health outcomes.

## Introduction

Human faces encompass a wealth of information about age, ethnicity, emotions, and health status.^1^ Distinct facial features can serve as indicators of various medical conditions and systemic diseases.^2^ For instance, Down syndrome can be identified by distinct features such as small eye fissures, a wide distance between the eyes, upward-slanting eyes, a flat facial profile, and a protruding tongue.^3^ However, the early detection of these phenotypic markers poses a significant challenge owing to the considerable variability in human facial features and the subtlety of some disease manifestations. This variability often necessitates the expertise and experience of healthcare professionals to make accurate assessments.^4^ Moreover, there is a pressing need for an effective, convenient, and cost-effective screening tool that can facilitate patient screening outside of hospital settings.^5^

In the contemporary healthcare era, marked by the integration of advanced digital technologies, artificial intelligence (AI) has catalyzed significant innovations.^6^^,^^7^^,^^8^ AI-assisted facial analysis is a promising new method for disease screening. This technology demonstrates remarkable potential in recognizing and interpreting facial attributes associated with various health conditions, offering a convenient and accessible preliminary screening method.^9^ While AI models offer valuable insights, it is essential to understand that they yield probabilistic assessments rather than definitive diagnoses. By providing a non-invasive, user-friendly, and cost-effective screening approach, AI-assisted facial analysis can contribute to early detection and prompt medical attention, particularly in low- and middle-income countries (LMICs).^10^ These regions often face a limited healthcare infrastructure and a shortage of medical professionals, leading to higher rates of medical complications and fatalities.^11^

AI-assisted facial analysis could bridge this gap by enabling earlier disease detection and management due to three key attributes. First, it is user-friendly and convenient, simplifying health assessments by utilizing facial images for analysis.^4^ Second, it is non-invasive and safe, prioritizes patient comfort, and minimizes risks by avoiding invasive procedures.^12^ Thirdly, it is cost-effective and affordable, requiring no expensive equipment or complex procedures, making it especially attractive for healthcare in resource-limited settings.^13^ These attributes establish AI-assisted facial analysis as a valuable tool in healthcare, facilitating comprehensive and continuous disease management. Notably, AI-assisted facial analysis is intended to complement professional medical evaluations, and its findings should be interpreted within the context of comprehensive clinical assessments. Moreover, ethical concerns regarding bias, fairness, and profiling remain critical.^14^^,^^15^ There is also a need for transparency, regulation, and alignment with societal expectations.^16^^,^^17^

Thus, we begin by outlining four distinct stages of a prototypical AI-assisted facial analysis technology pipeline (workflow of AI-assisted facial analysis technology). This is followed by a comprehensive examination of its main applications in disease screening (AI-assisted facial analysis for disease detection). Subsequently, we explore its potential to contribute to healthcare across other domains, including health monitoring, aiding treatment decision-making, and post-treatment follow-up, thereby facilitating comprehensive disease management (AI-assisted comprehensive management). However, these advances present technical and ethical challenges that demand careful consideration. We thoroughly and critically investigate these related issues and suggest potential future research directions (challenges and future directions). By providing a balanced perspective, we aim to highlight the potential challenges of AI-assisted facial analysis in healthcare.

## Workflow of AI-assisted facial analysis technology

**Figure 1:**
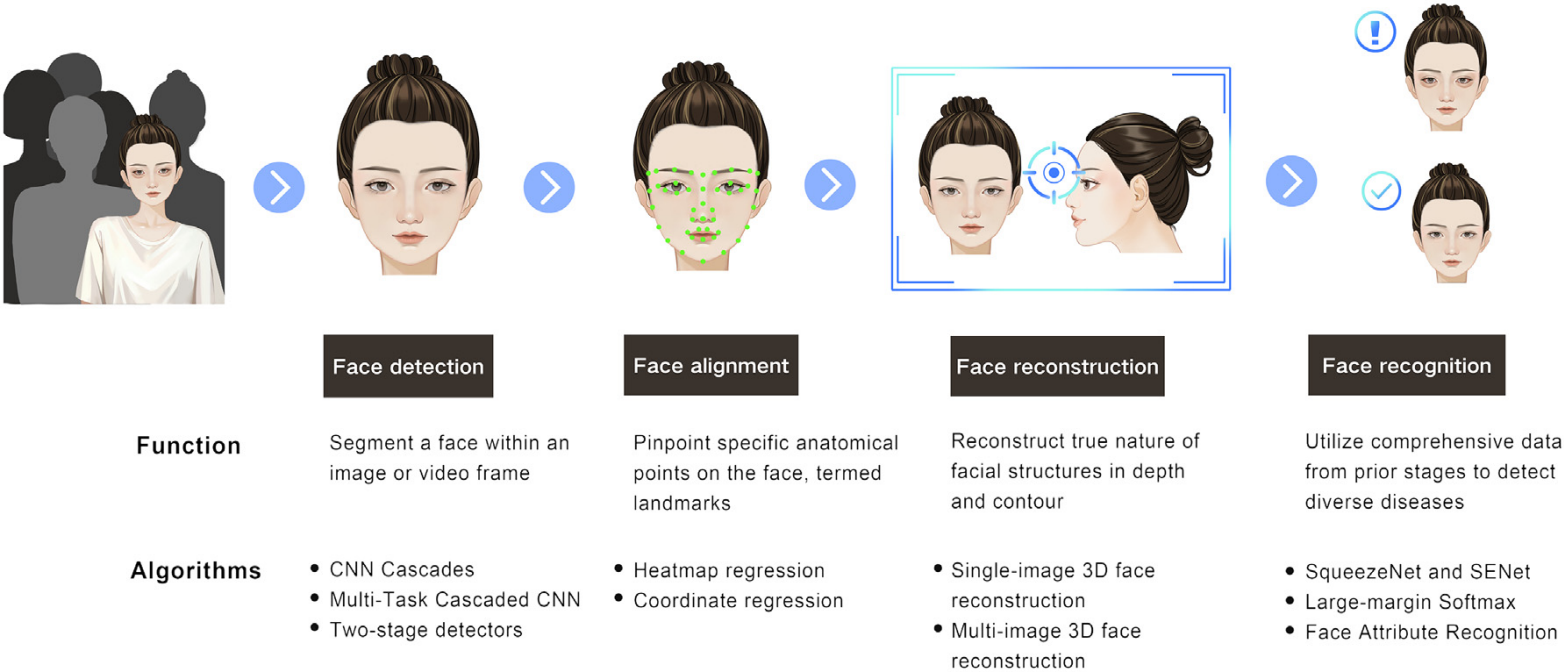
The workflow of AI-assisted facial analysis in disease detection There are four distinct stages of a prototypical AI-assisted facial analysis technology pipeline: face detection, face alignment, face reconstruction, and face recognition.

The convergence of computer vision and healthcare is changing the medical sector, particularly disease identification through AI-assisted facial analysis. This multifaceted process begins with face detection and alignment, potentially progressing to face reconstruction and ultimately culminating in face recognition (Figure 1). Each stage enhances the accuracy and efficiency of disease detection, thus shaping the future of healthcare.

### Face detection

Face detection is a critical component of facial processing applications. This involves locating and identifying faces in images or videos by filtering background noise and prioritizing faces as the main focus. Face detection techniques can be categorized into traditional methods and deep learning-based strategies. Traditional methods, such as those found in the OpenCV and Dlib libraries, utilize algorithms such as Haar cascades^18^ and Dlib’s histogram of oriented gradients with linear support vector machines.^19^

Deep learning methods significantly outperform traditional techniques. Convolutional neural network (CNN) cascades,^20^ multitask cascaded CNNs,^21^ and two-stage detectors, such as region-based CNN,^22^ represent some of the advances in this field. Additionally, anchor-free techniques like CenterFace^23^ and one-stage detectors like Single Shot MultiBox Detector^24^ have been developed for real-time detection and robust performance.

### Face alignment

Face alignment or facial landmark localization is essential for highlighting anatomical landmarks on the face. Techniques for facial landmark localization are primarily divided into heatmap regression and coordinate regression. Heatmap regression networks like Hour-Glass and High-Resolution Network^25^ predict likely landmark locations, while coordinate regression uses streamlined CNNs to precisely pinpoint facial landmarks.^26^ Enhanced methods such as HRNetv2,^27^ Practical Fast Landmark Detector,^28^ and Deep Adaptive Graph Network^29^ have further improved the accuracy of facial landmark localization, opening new opportunities.

### Face reconstruction

The depth and contours of facial structures are critically important. Disease screening based on facial features can be limited to a two-dimensional (2D) perspective. Therefore, three-dimensional (3D) face reconstruction is utilized to generate 3D facial models from 2D images. Recent breakthroughs in single-image 3D face reconstruction have shown promising results.^30^ For instance, direct volumetric CNN regression involves a straightforward CNN that transforms 2D images into volumetric depth maps.^31^ Multi-image 3D face reconstruction leverages multiple 2D images to aggregate shape information, handling occlusions and significant pose variations better than single-image reconstruction techniques.^32^^,^^33^

### Face recognition

The final stage in the AI-assisted facial analysis process is face recognition, which confirms an individual’s identity using data obtained from the previous stages. Beyond verification and identification, face recognition is also useful for attribute recognition, making it valuable in detecting a wide range of diseases.^34^ Advancements in network architectures have transitioned from early models such as AlexNet^35^ to lighter, more recent networks such as SENet.^36^ Improvements in loss function design, which are crucial for training deep neural networks, have also been noted. Techniques like CurricularFace^37^ enhance the performance of face recognition systems.

Face attribute recognition plays a key role in disease detection by identifying specific facial attributes. Multitask networks such as ANet^38^ and Partially Shared Multi-task Convolutional Neural Network with Local Constraint^39^ have been proposed for simultaneous attribute prediction, enabling the identification of diseases. Future improvements in AI-assisted facial analysis may enable earlier and more precise screening and advance healthcare outcomes.

## AI-assisted facial analysis for disease detection

**Figure 2:**
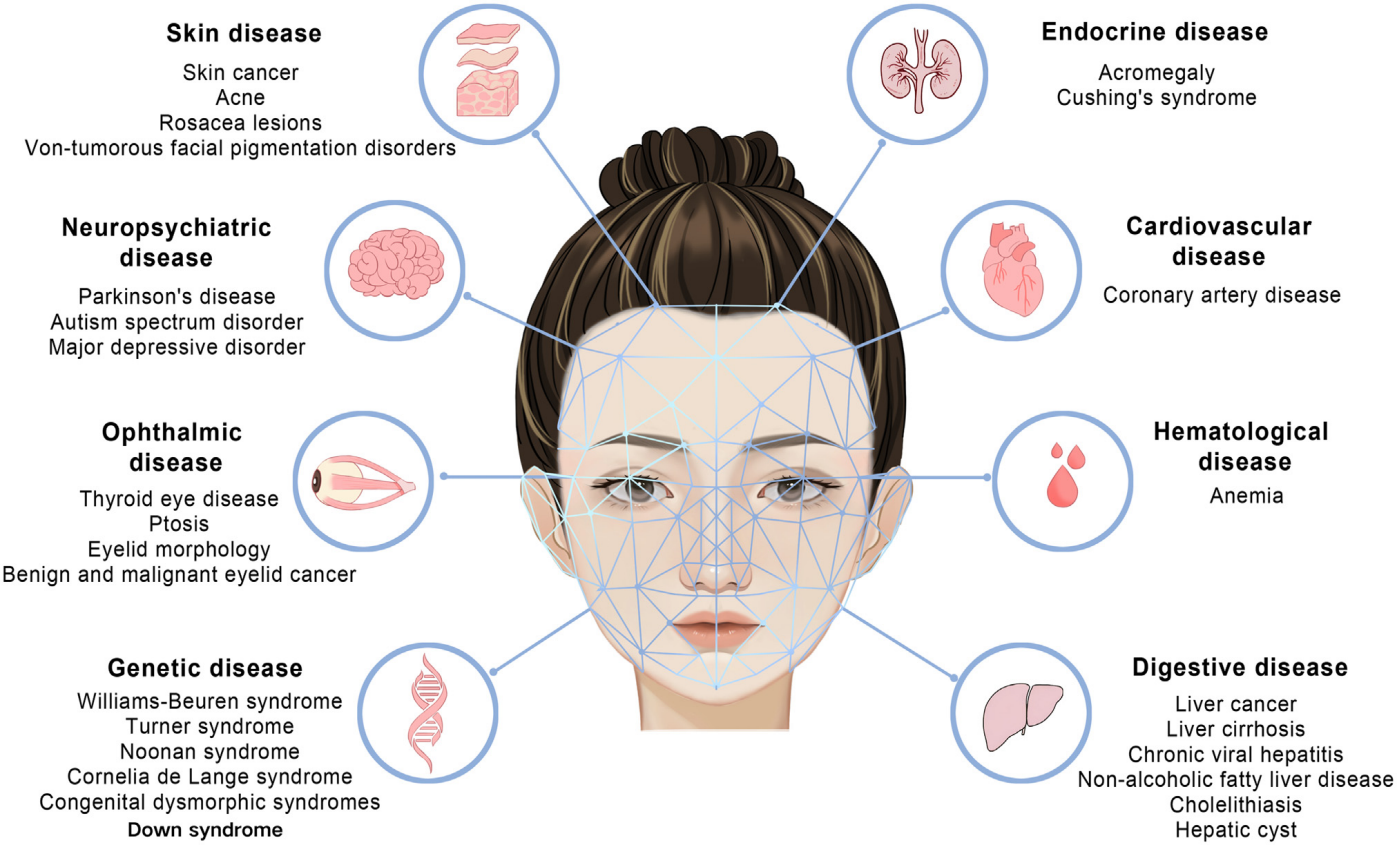
Diseases detectable by AI-assisted facial analysis Diseases detectable by AI-assisted facial analysis include skin diseases, neuropsychiatric diseases, ophthalmic diseases, genetic diseases, endocrine diseases, cardiovascular diseases, hematological diseases, and digestive diseases.

AI-assisted facial analysis has been used to screen a broad spectrum of diseases, as illustrated in Figure 2 and Table 1. For diseases with noticeable facial alterations, where diagnosis relies heavily on facial images, such as skin diseases, and where AI facial analysis achieves high accuracy, this tool provides valuable diagnostic support. In cases where facial changes are superficial indicators of underlying conditions such as cardiovascular disease, AI-assisted facial analysis serves as an effective screening tool to facilitate early detection. Our aim is to explore the potential of this technology to aid early disease detection, which could greatly improve patient health outcomes. This is especially beneficial for LMICs because (1) it is widely accessible, therefore enhancing global health equity; (2) it promotes early detection and improves chances of recovery; and (3) it can be used for self-monitoring and for increasing healthcare access.

**Table 1:** AI facial analysis for detecting diverse diseases

Study and year	Disease	Countries	Data type	No. of images	Compared with clinicians	Performance
**Genetic diseases**						

Porras et al.^40^	genetic syndromes	Argentina, Australia, Belgium, and 26 other countries	2D image (frontal)	2,800	no	accuracy: 0.88; sensitivity: 0.90; specificity: 0.86
Pan et al.^41^	Turner syndrome	China	2D image (frontal)	1,281	no	AUC: 0.95, 0.97, 0.96 (three scenarios); sensitivity: 0.97; specificity: 0.97
Yang et al.^42^	Noonan syndrome	China	2D image (frontal)	420	yes	AUC: 0.98; accuracy: 0.92
Latorre-Pellicer et al.^43^	Cornelia de Lange syndrome	Spain	2D image (frontal)	49	no	accuracy: 0.98
Mishima et al.^44^	congenital dysmorphic syndromes	Japan	2D image	108	no	accuracy: 0.86

**Skin diseases**						

Han et al.^45^	skin cancer	South Korea	2D image (frontal, side, and diagonal lines)	182,348	yes	AUC: 0.91; specificity: 0.77; sensitivity: 0.91
Wen et al.^46^	acne	China	2D image (half-face)	1,222	no	mean average precision: 0.54
Binol et al.^47^	rosacea lesions	US	2D image (frontal, half-side, looking up)	41	no	Dice coefficients: 0.90, 0.88 (two models)
Peng et al.^48^	non-neoplastic facial pigmentation disorders	Singapore	2D image (site of the facial lesion)	150	no	accuracy: 0.92

**Ophthalmic diseases**						

Karlin et al.^49^	TED	US	2D image (frontal)	2,166	yes	accuracy: 0.89; sensitivity: 0.93; specificity: 0.87; precision: 0.80; F1 score: 0.86
Huang et al.^50^	thyroid-associated ophthalmopathy	China	2D image (front, left, right, and nine eye positions)	21,840	no	AUC: 0.85; sensitivity: 0.80; specificity: 0.79; F1 score: 0.80
Lei et al.^51^	Graves’ orbitopathy	China	2D image (frontal)	943	no	AUC: 0.85
Hung et al.^52^	ptosis	US, China (Taiwan)	2D image (eye images)	832	yes	AUC: 0.986; sensitivity: 0.92; specificity: 0.88
Lou et al.^53^	ptosis	China	2D image (eye images)	135	yes	the ICCs between manual and automated measurements ranged from 0.934 to 0.971 (*p* < 0.01).
Tabuchi et al.^54^	ptosis	Japan	2D image (eye images)	1,276	yes	AUC: 0.90; accuracy: 0.83; sensitivity: 0.83; specificity: 0.83
Hui et al.^55^	benign and malignant eyelid cancer	China	2D image (frontal)	345	yes	AUC: 0.97; accuracy: 0.89; sensitivity: 0.93; specificity: 0.86
Chen et al.^56^	16 ophthalmic disorders	China	videos	3,652	no	AUC: 0.843, 0.859 (two settings)

**Endocrine diseases**						

Kong et al.^57^	acromegaly	China	2D image (frontal)	1,365	no	sensitivity: 0.96; specificity: 0.96
Kong et al.^58^	acromegaly	China	2D image and 3D video frames	2,148	yes	accuracy: 0.91
Wei et al.^59^	Cushing’s syndrome, acromegaly	China	2D image	14,544	yes	AUC: 0.96 (for both diseases)

**Neuropsychiatric diseases**						

Ali et al.^60^	PD	US	video (10–12 s)	1,812	no	AUC, 0.94; accuracy: 0.96; precision: 0.96; sensitivity: 0.94; F1 score: 0.95
Alam et al.^61^	ASD	–	2D image	2,840	no	accuracy: 0.95
Zhou et al.^62^	MDD	–	video (25 min)	150	no	mean absolute error/root-mean-square error: 6.20/8.28, 6.21/8.39 (for two datasets)

**Other diseases**						

Xiao et al.^63^	hepatobiliary diseases	China	2D image (ocular image)	3,550	yes	AUC: 0.74; sensitivity: 0.64; specificity: 0.73; F1 score: 0.68
Lin et al.^64^	CAD	China	2D image (frontal, profile, and top views)	6,229	yes	AUC: 0.73; sensitivity: 0.80; specificity: 0.54
Zhang et al.^65^	anemia	China	video (15 s)	316	yes	AUC: 0.84; accuracy: 0.82

ICC, intraclass correlation coefficient.

### Genetic disease

Advances in AI have enabled the recognition of facial features associated with genetic diseases. One study performed an analysis of 2,800 facial photographs from 1,400 children encompassing 128 different genetic diseases and 1,400 matched controls.^40^ Their AI model achieved an average accuracy of 88% for detecting these genetic diseases. This efficient technique offers a more accessible and affordable option for the early risk assessment of specialist referrals. Turner syndrome often presents with distinct facial deformities, such as a highly arched palate, low posterior hairline, and micrognathia. Researchers have developed a face recognition system specifically for detecting Turner syndrome.^41^ Similarly, Noonan syndrome, characterized by rapid eye movement and ptosis, can also benefit from AI-based screening.^42^ Furthermore, a smartphone app called Face2Gene was evaluated for screening Cornelia de Lange syndrome, and the correct detection was listed as the first prediction for 83.7%.^43^ Additionally, a study in Japan demonstrated the efficacy of Face2Gene in aiding clinical specialists in the identification of congenital dysmorphic syndromes.^44^ These tools offer accessible and affordable options for preliminary screening, potentially leading to earlier specialist referrals and interventions.

### Skin disease

Many skin conditions require visual detection, which is subjective and prone to error.^66^ Screening skin diseases in resource-limited areas is challenged by a lack of dermatologists and screening tools. The application of AI to the identification of skin diseases on the face has seen remarkable advancements. One study implemented region-based CNNs to determine the presence of skin tumor lesions in images and classified them as benign or malignant.^45^ Their research employed a dataset comprising 182,348 training images, 2,844 validation images, and 325 test images. The accuracy of the algorithm was comparable with that of the dermatologists (F1 score: 0.831 vs. 0.835; Youden index score: 0.675 vs. 0.671). Acne vulgaris is a prevalent skin disorder that primarily affects adolescents and is characterized by papules, nodules, and facial cysts. Researchers have devised CNNs that were subsequently integrated into a free WeChat mini program to screen and classify the severity of acne.^46^ Rosacea is a treatable but chronic condition characterized by facial flushing, erythema, telangiectasia, and edema. A computer-aided screening system known as Ros-NET was developed to effectively identify rosacea lesions.^47^ Additionally, the detection of non-neoplastic skin pigmentation disorders was addressed through deep learning models.^48^ Therefore, AI-assisted facial analysis can improve the accuracy of detecting several skin diseases, which is expected to facilitate early intervention.

### Ophthalmic disease

Ophthalmic disease management in low-resource settings is constrained by insufficient ophthalmic services and screening equipment.^67^ Many eye conditions, such as orbital and eyelid diseases, exhibit changes in the ocular and facial regions, providing a foundation for the application of AI-assisted facial analysis.^68^^,^^69^ Thyroid eye disease (TED) is the most common orbital disease and is characterized by exophthalmos, strabismus, and eyelid retraction.^49^ An ensemble neural network model was developed, using a dataset of 1944 photographs for training, 344 for performance metric calculations, and 222 for assessment.^49^ The model demonstrated an accuracy of 0.892. Additionally, the ResNet-50 model was employed to detect thyroid-associated ophthalmopathy based on external ocular images,^50^ and facial expressions were also explored to detect Graves’ orbitopathy.^51^ Ptosis can affect vision and is a symptom of ocular myasthenia. AI-based systems capable of automatically screening ptosis and measuring eyelid morphology using external ocular images were developed.^52^^,^^53^ The iOS app was devised to detect ptosis via smartphone-captured facial images.^54^ Eight CNNs were employed to automatically detect benign and malignant eyelid tumors from facial images, achieving an area under the curve (AUC) of 0.966.^55^ Furthermore, a smartphone-based deep learning system was developed to detect visual impairment in young children.^56^ In summary, AI-assisted facial analysis offers a promising solution for the early detection of eye diseases, especially orbital and eyelid diseases.

### Endocrine disease

Endocrine diseases often go undiagnosed due to the lack of routine screening and awareness, with delayed diagnosis leading to severe complications.^70^ Patients with Cushing’s syndrome and acromegaly exhibit distinct facial characteristics. One study focused on the detection of acromegaly with facial features, including an enlarged nose, prominent mandibular and zygomatic arches, thickened lips, and facial soft tissue swelling. Various machine learning techniques were harnessed to identify acromegaly using facial images,^57^ achieving a sensitivity of 96% and a specificity of 96%, thereby facilitating automated acromegaly detection. In subsequent research, a novel deep learning-based model was developed to not only screen acromegaly but also classify its severity, expanding the scope of AI facial analysis.^58^ AI’s impact extends to other endocrine disorders, including Cushing’s syndrome, with an estimated incidence of hypercortisolism being 3 per million.^59^ A deep learning-based model was proposed for identifying Cushing’s syndrome and acromegaly from facial images, demonstrating an AUC of 0.96 for both diseases.^59^ The results were comparable in sensitivity and specificity to those of a physician. Hence, AI-assisted facial analysis can play a crucial role in identifying the signs of endocrine disorders, enabling prompt management and improving long-term health outcomes.

### Neuropsychiatric disease

The shortage of mental health professionals and prevailing stigma significantly hinder the early diagnosis of neuropsychiatric disorders in resource-constrained settings.^71^ Conditions such as autism spectrum disorder (ASD), major depressive disorder (MDD), and Parkinson’s disease (PD) often display symptoms of reduced or abnormal facial expressions, rendering them suitable for detection through AI-assisted facial analysis. One study used a dataset of 1,812 videos from 604 individuals to develop a model for PD detection, achieving an accuracy of 0.956, precision of 0.958, and AUC of 0.940.^60^ Similarly, the efficacy of AI-assisted facial analysis in accurately detecting ASD and MDD was also demonstrated.^61^^,^^62^ These investigations underscore the potential of AI-assisted facial analysis for enhancing and expediting the early identification of neuropsychiatric disorders.

### Other diseases

AI-assisted facial analysis is promising for the detection of other conditions. One study utilized external ocular images for the automatic detection of hepatobiliary diseases, achieving an AUC of 0.74 (95% confidence interval 0.71–0.76).^63^ Notably, among the included hepatobiliary diseases, the model demonstrated the best performance for liver cancer and liver cirrhosis. Moreover, the feasibility of employing facial images to detect coronary artery disease (CAD) was confirmed through deep learning models.^64^ The algorithm for detecting CAD exhibited a sensitivity of 0.80 and a specificity of 0.54 in the test group. Additionally, multiple models capable of predicting anemia were developed.^65^ The accuracy of the image level was 82.37%, and the AUC of the image level was 0.84. In summary, the integration of AI-assisted facial analysis presents a promising avenue for simplified and efficient detection of diverse medical conditions.

## AI-assisted comprehensive management

**Figure 3:**
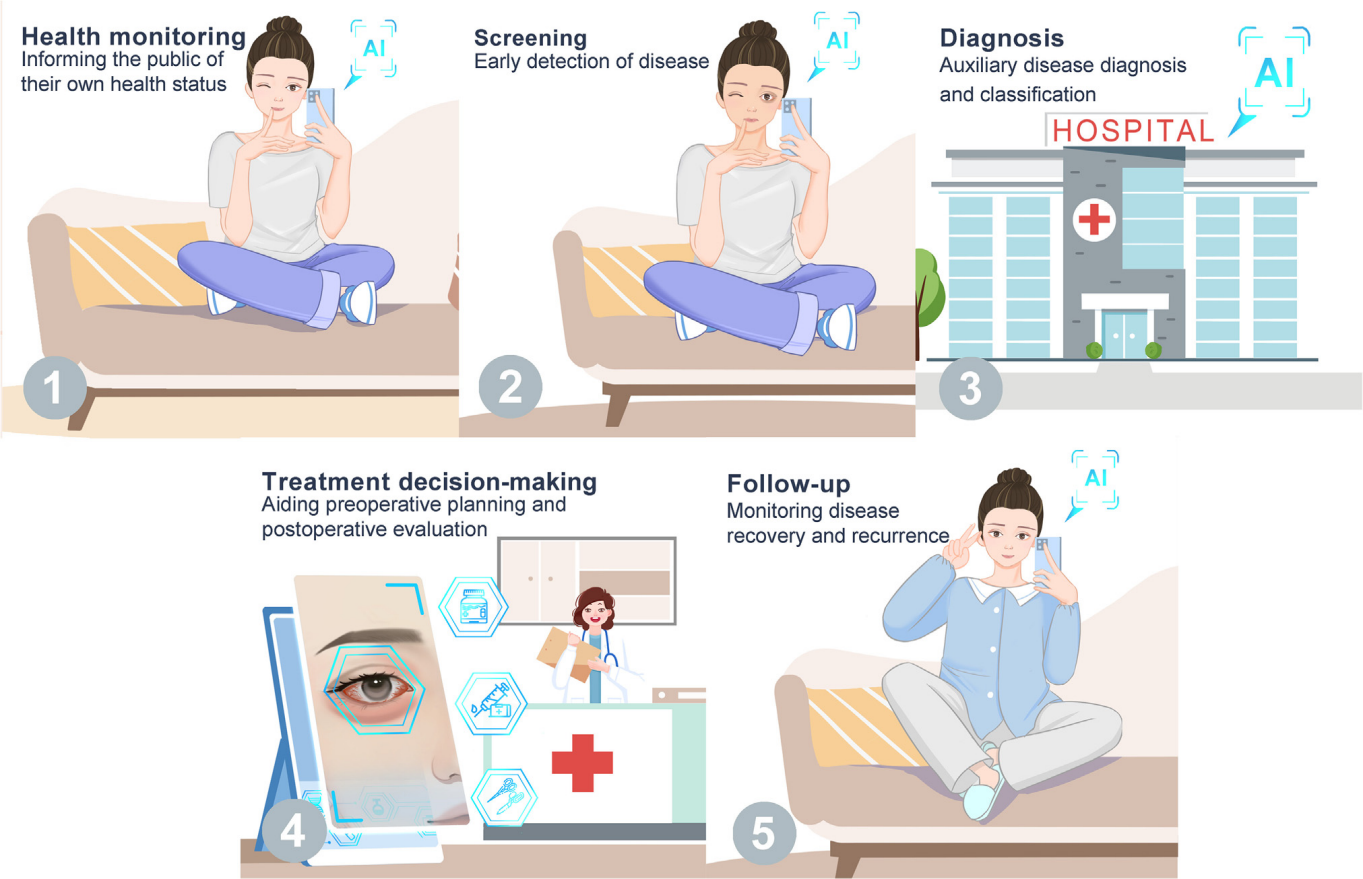
Comprehensive AI-assisted disease management This includes five aspects: health monitoring, early screening, disease diagnosis, treatment decision-making, and follow-up.

AI-assisted comprehensive management extends beyond disease screening to health monitoring, treatment decision-making, and follow-up. It aims to provide a holistic approach to disease management (Figure 3). Although AI has yet to be applied to every stage of disease management for a single disease, there are applications for each stage across various diseases.

### Empowering health monitoring

AI-assisted facial analysis can also be used for health monitoring. Personal attributes such as age, sex, and physique can be identified from facial images. For instance, the GRA Net utilizes facial images to predict age and sex, aiding in detecting premature facial aging.^72^ Xia et al. developed CNNs trained on non-invasive 3D facial images of 5,000 Han ethnic Chinese individuals, achieving an average difference between predicted and chronological/perceived age of ±2.8 and 2.9 years, respectively.^73^ ConstitutionNet was proposed to classify different constitutions in traditional Chinese medicine based on facial images, assisting in determining the most effective personalized conditioning program for individuals.^74^ Tay et al. developed models for real-time monitoring of nutritional status, enabling clinicians to remotely monitor patients and deliver necessary interventions.^75^ These applications provide individuals and healthcare providers with valuable information for maintaining health and preventing disease.

### Aiding treatment decision-making

Efforts have been made in preoperative planning and postoperative outcome assessments in treatment decision-making. For preoperative planning, Van Brummen et al. facilitated the measurement of the marginal reflex distance (MRD), which is used to evaluate eyelid and soft tissue position.^76^ Additionally, a specialized smartphone application for MRD and levator muscle function measurement was developed to enhance user convenience. Song et al. introduced a clinical decision model to aid in making surgical decisions based on multidimensional facial imaging data.^77^ For pain assessment, ResNet-18 was trained to assess pain intensity based on facial expressions, assisting vulnerable groups in objectively assessing pain intensity and helping doctors tailor treatments by gauging pain responses.^78^

AI-assisted facial analysis is valuable for evaluating postoperative outcomes. Lou et al. devised a method to automate the measurement of eyelid morphology before and after surgery, minimizing human error.^53^ Bahçeci Şimşek and Şirolu utilized the DLIB-ML toolkit, which contains various image processing and machine learning power detectors, to create an automated approach for evaluating the outcome of Muller’s muscle-conjunctival resection surgery.^79^ Hidaka et al. designed an esthetic evaluation tool for mandibular reconstruction based on facial images.^80^ Similarly, Zhai et al. developed the Beautynet system to preoperatively predict facial beauty outcomes, enabling personalized surgery plans.^81^ Thus, AI-assisted facial analysis provides more objective and efficient evaluations compared to manual assessments, producing compelling evidence to aid therapeutic implementation.

### Facilitating disease follow-up

AI-assisted facial analysis has the potential to greatly enhance disease follow-up by providing detailed and objective information regarding a patient’s progress and response to treatment. AI systems track changes in a patient’s facial features and expressions by comparing facial images captured at different intervals. For example, Hossain and Muhammad proposed an e-healthcare framework in which mobile devices such as smartphones capture images and medical data, which are then sent to the cloud for processing.^82^ The results are shared with healthcare professionals, and in cases of negative emotions such as pain, caregivers can be alerted for patient support. In dermatology, AI facial technology can monitor the progress of skin conditions, such as acne, eczema, or psoriasis.^83^ This enhances patient engagement and can improve adherence to treatment plans.

To conclude, AI-assisted facial analysis enables effective disease management. Individuals can use the AI-based software on their smartphones to monitor their health. If potential health issues are detected, the software identifies patients who may require specialist care, thereby facilitating early intervention. Doctors can also use this technology after diagnosis, and AI models can aid in creating treatment plans and evaluating surgical results using patient photographs. After treatment, patients can upload their facial images to the software for follow-up, which enables doctors to collect data and track recovery. Therefore, AI-assisted facial analysis provides an integrated solution that combines technology, healthcare, and patient well-being.

## Challenges and future directions

**Figure 4:**
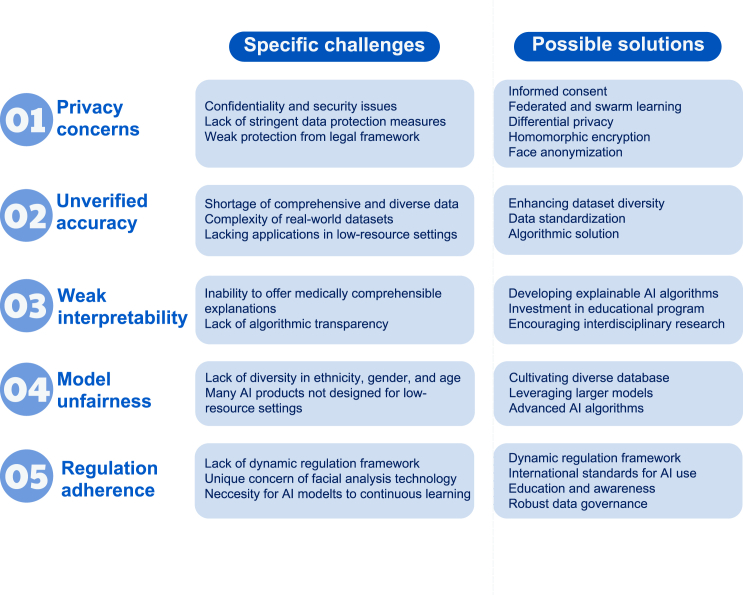
Challenges and future directions They include privacy concerns, unverified accuracy, weak interpretability, model unfairness, and regulation adherence.

Although AI-assisted facial analysis offers significant opportunities, challenges must be addressed to ensure its effective and ethical implementation. These include privacy concerns, unverified accuracy, weak interpretability, model unfairness, and regulatory adherence (Figure 4). In this section, we delve into each challenge, propose viable strategies to address them, and highlight the importance of future research. Such measures are crucial not only for the integration and acceptance of technology in healthcare environments but also for building trust among patients and practitioners. Collaborative efforts among technologists, clinicians, ethicists, and policymakers are vital for addressing these challenges and harnessing the full potential of AI-assisted facial analysis in healthcare.

### Privacy concerns

Privacy concerns are critical challenges when deploying AI-assisted facial analysis for disease detection. The collection, storage, and analysis of facial data raises significant concerns about the confidentiality and security of individuals’ personal information.^84^ Without stringent data protection measures, there is a risk of unauthorized access to sensitive health information, which can lead to misuse, discrimination, and a breach of trust between patients and healthcare providers.^85^ Malicious individuals may exploit user profiles on social media platforms to develop applications capable of identifying potential health risks without user consent. This could result in the unwarranted disclosure of private health information and expose users to targeted advertising for medical treatment or services, infringing upon their privacy and autonomy. Such activities may also lead to psychological distress or social stigma among affected individuals. This issue is compounded in areas where legal frameworks for data protection are weak or nonexistent, thereby increasing the vulnerability of individuals to privacy violations.

It is important to balance these concerns with the urgent need for AI-assisted facial analysis and the substantial value it brings to healthcare. Privacy concerns must be considered when deploying AI-assisted facial analysis. First, robust informed consent is critical.^86^ Individuals must have a clear understanding of what consent entails, the associated risks of sharing their facial data, and their rights regarding the use and disclosure of disease detection. Second, federated learning enables AI models to train on decentralized medical data without requiring the data to be centralized, thereby mitigating the risk of data breaches.^87^^,^^88^ Building upon this, swarm learning extends this concept by combining edge computing and blockchain-based peer-to-peer networks, enabling global collaboration on medical data while preserving privacy and eliminating the need for a central coordinator.^89^ Third, leveraging privacy-enhancing technologies throughout the AI life cycle is recommended. During training, differential privacy is employed to prevent the model from memorizing individual patient data while still allowing it to learn generalizable patterns.^90^ During inference, auxiliary patient data are encrypted before transmission to the model and decrypted and processed within a secure, isolated environment. Fourth, homomorphic encryption can be utilized to allow data to be processed in encrypted form, ensuring that data privacy is maintained during analysis.^91^ Furthermore, adopting face anonymization techniques can help maintain the utility of AI-assisted facial analysis for disease detection while safeguarding individual identities.^92^ It is important to note that, while these techniques protect data during training, they may not address all privacy concerns in deployment, particularly when making individual health inferences. In addition, raising public awareness about the potential misuse of facial data can empower users to take precautions when sharing images online. Collaborative efforts among governments, technology companies, and international organizations are essential for developing and enforcing standards that prevent the exploitation of AI technologies for malicious purposes.

### Unverified accuracy

The unverified accuracy of AI-assisted facial analysis for disease detection in low-resource settings presents a complex challenge closely tied to the complexities of datasets and the shortage of comprehensive and diverse data crucial for developing effective AI models. These settings often lack the advanced imaging technology and infrastructure needed to gather, store, and process the extensive, high-quality data required.^93^ Existing datasets are usually small and not representative of the wide range of racial backgrounds or medical contexts needed for AI to be truly effective.^94^ Additionally, the variability in real-world data capture conditions, including differing camera specifications, poor lighting conditions, blurred facial images, and deviations in facial angles, further complicates the dataset and impacts the precision and reliability of AI models.^67^ Thus, the accuracy rates reported in the literature may not translate to real-world settings. This variability renders it particularly challenging to deploy these technologies in low-resource areas to broaden access to healthcare facilities through mobile AI-assisted facial analysis applications.^52^

To overcome the challenge of low model accuracy owing to dataset complexities and data shortages, a multifaceted strategy is required. Enhancing dataset diversity and volume through international collaboration and inclusive data collection efforts is fundamental.^95^ Engaging a broad spectrum of participants from various ethnic backgrounds and medical conditions can enrich datasets, ensuring that AI models are trained and validated on comprehensive and representative data. Embracing advanced learning methodologies, such as transfer learning,^96^ few-shot learning,^97^ and self-supervised learning,^98^ can mitigate the impact of limited data by enabling models to learn effectively from small datasets. Other algorithmic solutions, such as domain adaptation and robustness enhancement strategies, can further refine model performance under diverse conditions.^34^^,^^99^ However, these methods may not fully compensate for the fundamental limitations in data representativeness and may introduce new uncertainties. Standardization of data acquisition processes is another critical measure that involves establishing uniform protocols and quality control mechanisms. For images with suboptimal quality, sequential enhancement techniques can be employed to improve image fidelity. Techniques such as facial image relighting adjust lighting conditions while preserving facial features, mitigating the effects of poor illumination.^100^ Deblurring algorithms can reduce or eliminate motion blur by learning from large datasets of clear and blurred images, thereby restoring image clarity.^101^ Facial image registration, also known as face normalization, calibrates images with angular deviations to align faces directly with the camera, providing a more comprehensive view of facial features.^102^ By implementing these solutions, the dual challenges of dataset complexity and data shortage can be addressed. Finally, AI research incorporating images captured by patients or users using smartphones as external test datasets is highly recommended. Including such real-world data can better assess the robustness and generalizability of models in practical applications, ensuring that they perform effectively when deployed outside of controlled environments.

### Weak interpretability

Weak interpretability in AI models, especially in facial image analysis for disease detection in low-resource settings, poses a significant challenge to the acceptance and trust of AI-driven screening.^103^ Advanced techniques, such as class activation mapping (CAM) and attention-based mechanisms, despite providing visual insights, often fail to offer medically comprehensible explanations, especially without clear causal links between facial features and diseases. Medicine clearly understands the facial changes that many skin and genetic diseases cause. However, for conditions such as coronary heart disease, better explanations from medical mechanisms are needed; otherwise, relying solely on detection accuracy makes it difficult for healthcare professionals and patients to fully understand and trust AI decisions.^104^^,^^105^ The lack of algorithmic transparency not only hinders clinical integration but also limits the potential of AI to contribute to medical research by obscuring insights into disease pathophysiology.^106^^,^^107^ Consequently, enhancing interpretability is essential for building confidence in AI within healthcare, ensuring that AI tools are both reliable and comprehensible, particularly in settings where the need for accessible and understandable screening solutions is critical.

To overcome the challenge of weak interpretability in AI models, it is crucial to develop explainable AI algorithms.^108^ Enhancements in CAM and attention-based mechanisms should aim not only to provide visual evidence of AI’s conclusions but also to translate these insights into explanations that are clear and meaningful for healthcare professionals and patients.^109^ This involves collaborative efforts between AI developers, data scientists, and medical experts to refine these techniques, ensuring that they yield transparent and clinically relevant outcomes. Additionally, investing in educational programs that equip healthcare professionals with the knowledge to understand and interpret AI-driven disease detection can foster trust and acceptance in clinical settings.^110^ This includes exploring why diseases exhibit characteristic manifestations on the face by investigating at the anatomical, pathophysiological, and other mechanistic levels. Not only can this better explain AI facial analysis in disease detection from a medical perspective, but it may also serve as a window to discover new pathogeneses of diseases. Such initiatives could significantly improve the usability of AI in healthcare, particularly in low-resource environments, by providing tools that are reliable and easily integrated into clinical workflows.

### Model unfairness

Model unfairness in AI-assisted facial analysis for disease detection arises when AI models perform differently across demographic groups due to biases in their training datasets, a situation exacerbated by a lack of diversity in ethnicity, sex, and age.^111^ This can result in reduced accuracy for underrepresented populations, exacerbating health disparities and leading to potentially mistaken or missed detection.^104^ This issue is highlighted by the remarkable differences in facial characteristics among ethnic groups, suggesting the need for more inclusive datasets and the creation of ethnicity-specific datasets to improve screening accuracy.^112^^,^^113^ Furthermore, AI products, often designed in East Asia and Western contexts, may not effectively translate to low-resource environments without adjustments to align with local healthcare needs.^114^ This reliance on data from high-resource areas overlooks the diverse disease spectrum and genetic diversities in less affluent regions, potentially exacerbating health inequities and limiting the global applicability of AI in healthcare.

To address these issues, developing AI systems with built-in fairness mechanisms is crucial. Despite the evolution of advanced AI models, such as large language models,^115^ generative pre-trainedtransformers,^116^^,^^117^ and Segment Anything Model,^118^ which have benefitted from extensive training on big data, persistent biases underscore the necessity of international collaboration among healthcare entities. This collaboration aims to cultivate diverse datasets with significant representation from low-resource settings to address the critical challenge of bias and ensure equitable, unbiased, and effective global healthcare solutions. To address this, leveraging larger models alongside varied datasets has emerged as a promising approach to mitigate biases and foster fairness within AI-based disease screening.^119^ Furthermore, contemporary research is delving into strategies such as unbalanced training,^120^ attribute obfuscation,^121^ and domain adaptation.^118^^,^^122^ Nevertheless, eliminating bias remains challenging, and ongoing efforts are required to understand and address the underlying causes of model disparities.

### Regulation adherence

The challenges of regulatory adherence in deploying AI-assisted facial analysis for disease detection highlight this complex landscape. The swift evolution of AI technologies often surpasses the pace at which regulatory frameworks are established, creating a gap in ensuring the ethical and fair use of the technology.^123^ In addition, regulations vary across jurisdictions, creating complexities in ensuring compliance, especially in international applications. The unique concerns of AI-assisted facial analysis, such as privacy and consent, also require stringent data governance frameworks that are challenging to implement, especially in low-resource environments.^124^ Furthermore, the necessity for AI models to continuously learn and adapt to new data complicates adherence, as models may deviate from their original validation and require ongoing oversight.^125^

Solving the challenges of regulatory adherence involves several key steps. First, creating dynamic regulatory frameworks that can quickly adapt to new technological advancements ensures that guidelines remain relevant and enforceable as AI evolves.^126^ This requires close collaboration between technology developers, regulators, and ethicists to anticipate future trends and address them proactively. Second, establishing international standards for AI use in healthcare can help manage differences across jurisdictions, ensuring a more uniform approach to ethical considerations and privacy protection.^127^ Recently, Annex III of the EU AI Act specified high-risk AI systems, including those for biometric identification.^128^ While facial data-based disease detection AI is not explicitly listed, such systems must still rigorously protect data privacy and security. Title II, Article 5, section (dc) limits emotion recognition systems, although medical use is exempted. Therefore, facial data, which are rich in biological and emotional information, require careful consideration in all applications.

Additionally, enhancing education and awareness of data privacy and consent, especially in low-resource settings, is crucial. This could involve training programs for healthcare workers and public awareness campaigns to inform patients about the rights and implications of AI in their care. It is encouraging to see regulatory frameworks being initiated, including the GDPR (2018) and AI Act (2021) in the EU as well as China’s Data Security Law and Personal Information Protection Law (2021).^129^ However, the underlying principles remain subject to ongoing debate and are influenced by cultural contexts. Therefore, establishing robust data governance, along with continuous monitoring and reevaluation of AI systems, will be essential for maintaining compliance over time as models evolve and adapt.^130^ This includes setting up mechanisms to regularly review and adjust AI models to ensure they meet regulatory standards and continue to operate ethically.^131^ Finally, it is crucial to differentiate medical uses from surveillance applications, as the context of use significantly shapes the ethical and practical implications. While public surveillance and law enforcement applications of facial recognition raise unique concerns, these challenges are distinct from those encountered in medical contexts, where the focus is on patient care and health outcomes.^132^ By tackling these areas, we can work toward a solution that ensures that AI is used responsibly and ethically in healthcare, benefitting patients globally while adhering to regulatory standards.

## Discussion

AI-assisted facial analysis has significant potential in healthcare. This technology, with its non-invasive, user-friendly, and cost-effective attributes, is poised not only to advance disease screening but also to seamlessly integrate with telemedicine frameworks, enhancing disease management.^133^ The performance of the AI model has proven to be comparable to that of board-certified specialists and surpassed that of physicians in certain studies.^45^ This evidence supports the potential reliability of AI-assisted facial analysis in settings lacking specialist expertise. Smartphone applications have been developed for screening genetic disorders,^43^ detecting ptosis,^54^ and identifying visual impairment in young children.^56^ These applications have indeed improved screening efficiency, helping patients understand their health status more conveniently and achieving early detection of certain diseases. By improving the accessibility of health assessments, these applications conveniently help individuals understand their health status, which is crucial for preventing disease progression and improving health outcomes at the population level. The widespread implementation of these technologies holds promise for enhancing public health by facilitating early diagnosis, reducing healthcare disparities, and strengthening disease management strategies.

The ethical implications of using this technology should be fully considered before deployment, with particular attention paid to its application in specific contexts.^134^ These include disparities related to digital access that reflect health and social inequalities across populations based on sex, education, income, and rural/urban location. Individuals facing barriers to healthcare also tend to be digitally excluded. Therefore, AI-assisted facial analysis should be designed and implemented with an equal lens to benefit those who need it the most. This necessitates algorithms with higher sensitivity to detect as many patients with the disease as possible. Moreover, it requires the patients themselves, their families, and even neighbors and friends to have access to smartphones to conveniently utilize this technology. Challenges include instituting stringent privacy protections, expanding and diversifying datasets to accurately represent all patient demographics, enhancing the interpretability of AI models, eliminating potential biases within AI applications, and meeting regulatory standards.^40^ Collaborative efforts involving governments, non-governmental organizations, technology companies, educational services, and local communities will be essential in tailoring these technologies to the unique needs of different regions, including addressing linguistic and cultural considerations.^135^^,^^136^^,^^137^ These partnerships are crucial not only for ensuring the technology’s acceptance and effectiveness but also for building local capacities for its maintenance and advancement, ensuring its sustainable integration into healthcare systems. Future research should focus on refining AI algorithms and increasing the range of diseases detectable by AI to ensure the broad applicability and effectiveness of the technology in diverse clinical and cultural contexts.^52^^,^^75^

Overcoming these challenges will pave the way for AI-assisted facial analysis to make a profound impact on healthcare.^83^ It promises to improve the accessibility and efficiency of healthcare, particularly benefitting those in LMICs, where such advancements could dramatically change healthcare delivery. In these regions, the technology’s ability to provide high-quality care with minimal resources could improve medical practices, making it a critical component in the global effort to enhance healthcare outcomes and achieve health equity.^138^ Continued innovation, guided by ethical considerations and collaborative efforts, will enable this technology to contribute meaningfully to global health goals, ultimately improving access to quality healthcare for all.

## Acknowledgments

We thank all of the investigators and participants in this study. This study was supported by the 10.13039/501100001809National Natural Science Foundation of China (82071003, 82271122, and 82388101); the Science and Technology Commission of Shanghai (20DZ2270800 and 23DZ2302200); the Shanghai Key Clinical Specialty, Shanghai Eye Disease Research Center (2022ZZ01003); and the Clinical Acceleration Program of Shanghai Ninth People’s Hospital, 10.13039/501100008233Shanghai Jiao Tong University School of Medicine (JYLJ202202). We also thank VoxelCloud Inc. for their support.

## Author contributions

C.L., K.D., S.S., Z.W., R.B., and X.Y. drafted the manuscript. All authors contributed to the conceptualization, analysis, and editing of the manuscript.

## Declaration of interests

The authors declare no competing interests.
